# The I. H. Association

**Published:** 1881-09

**Authors:** T. F. Pomeroy

**Affiliations:** Jersey City


### THE INTERNATIONAL HAHNEMANNIAN ASSOCIATION.

*Editor of* THE HOMŒOPATHIC PHYSICIAN:

I desire to express my great satisfaction with the July number of your journal; albeit it is but the report of the proceedings of our newly-formed Association—really its first working session, and that under great disadvantages. I am reminded of the old-time homœopathic journals, like the *American Review,* in its perusal, and am brought back to my early experiences and observations of homœopathic therapeutics, when the greatly-despised and ridiculed doses, "such little pills as a homœopathic doctor would bring," *would* work such wonderful cures.

It would perhaps be invidious to be more specific, although I might, as to the papers that were there presented, as they all of them had the old flavor of honest conviction, and of a truthful application of means to an end, of cause to effect.

The International Hahnemannian Association has no need to be ashamed of its beginning work as thus manifested through the columns of your journal, but quite the reverse. If it shall continue thus to exhibit the results of the application of the "strict inductive method of Hahnemann" to the profession, it has before it the continuance of the great work that was begun, and for many years continued after its first introduction into this country. I may truthfully say it will be a revival of that work, from which homœopathy on this continent received its first impulses, and through which it has gained its firm establishment here in spite of the degeneracy and retrogression that has apparently characterized its later years. I feel we are to be congratulated that homœopathy still lives, although perhaps a majority of those practicing under its law have measurably forgotten, as they have quite ignóred, those "strict inductive methods" under which alone homœopathic success is assured.

An adherence to the good old usage of only fully-proved agents in our therapeutics is quite as important and binding as is the avoidance of the methods and the crude agencies that belong to the allopathic and eclectic schools of medicine. With such therapeutic means as we now possess in the materia medica of our school, with its fully proved drugs, and with the numerous and varied helps in the study of it, no excuse is left for a departure from that already time-honored and reliable usage. I am glad, therefore, that the papers that were presented to our Association, as published by you, give no evidence of a sanction or indorsement of a contrary course, by a recital of cures attempted or claimed from the use of agents that have never been proved according to the methods laid down by Hahnemann. Surely, thus only may we lay claim to entire consistency, and assert the maintenance of our integrity.

The question of dosage, *i. e.*, of high or low attenuation, is one of comparatively minor importance, and about which there need be no controversy. The adaptation of the similar remedy must ever maintain the supremacy over the question of the dose; certainly until a law governing the dose is made manifest. Happily, this is a matter that will regulate itself, for, with the greater accuracy in the selection of the simillimum comes the necessity for *the* minimum dose, for the particular case under treatment; thus ensues a recognition of individualization, a cardinal principle of our therapeutics, as to the dose as well as to the selection of the similar remedy. This is a happy feature of our system of therapeutics, and our Association need give itself no concern nor make any attempt to regulate this question, as it has no prescribed rule of action in this direction. The faithful application of Hahnemann's strict inductive methods will accomplish all that can be desired by its members to this end.

The use of more or less attenuated, if you please, of potentized, remedies follows as a legitimate corollary of the homœopathic law of cure. The rest must be left, *ex necessitate*, to the intelligence and judgment of the prescriber; the degree of excellence in both of these elements that he has attained, will be the measure of his consistency as of his success. Our Association has not assumed, much less asserted that none but high dilutionists are consistent homœopathists, although the attempt has been made to thus falsely represent it to the profession. Such an assumption would be as absurd and as untruthful as the assertion that the use of remedies above the 30th is a violation of homœopathic principles, being, it is said, equivalent to the use of none at all; this has been openly asserted, and the maintenance of the statement attempted. But the facts are against both assumptions, and *facts* will ever rule supreme.

T. F. POMEROY.

